# Vital Exhaustion in Hospitalized Patients with Cardiovascular Disease: Associations with Anxiety and Insomnia—A Cross-Sectional Study

**DOI:** 10.3390/jcdd13070302

**Published:** 2026-07-01

**Authors:** Panagiota Aroniada, Vasiliki Tsoulou, Andriana Maggita, Athanasia Tsami, Maria Polikandrioti

**Affiliations:** Department of Nursing, University of West Attica, 122 43 Egaleo, Greece

**Keywords:** vital exhaustion, anxiety, insomnia, cardiovascular disease

## Abstract

Introduction: Vital exhaustion (VE), characterized by fatigue, irritability, frustration, and hopelessness, has been associated with the development and progression of cardiovascular disease (CVD) and is considered a maladaptive response to chronic stress. This study aimed to explore the association of VE with anxiety and insomnia in hospitalized patients with CVD, as well as the demographic and clinical characteristics associated with VE. Materials and Methods: A cross-sectional study was conducted including 200 hospitalized patients with CVD. VE was assessed using the Maastricht Questionnaire (MEQ); anxiety, using the Zung Self-Rating Anxiety Scale (SAS); and insomnia, using the Athens Insomnia Scale (AIS). Additionally, a structured questionnaire on clinical and demographic variables was utilized. Results: The majority of the 200 participants were men (60.7%) and aged 71–80 years (40%). The mean VE score was 21.4 ± 10.1 within score range from 0 to 40. VE was significantly associated with gender (*p* = 0.002), age (*p* = 0.006), occupation (*p* = 0.006), type of CVD (*p* < 0.001), comorbidity burden (*p* = 0.001), and perceived importance of written health information (*p* = 0.015). Higher VE scores were positively correlated with both anxiety and insomnia (*p* < 0.05). Conclusions: VE is closely associated with anxiety and insomnia and is influenced by key demographic and clinical factors in hospitalized patients with CVD. These findings highlight VE as a clinically relevant condition and identify distinct high-risk patient groups that may benefit from targeted assessment and management strategies.

## 1. Introduction

Vital exhaustion (VE), characterized by persistent fatigue, demoralization, irritability, and frustration, was first introduced by Appels et al. in 1987 and conceptualized as a maladaptive psychological response to chronic and uncontrollable stress [1].

Its clinical relevance in cardiovascular research is well-established, with evidence demonstrating that elevated VE is associated with an increased risk of myocardial infarction and adverse cardiac outcomes. Beyond its association with incident events, VE has been proposed as a prodromal state of acute coronary syndromes, characterized by a gradual decline in energy and functional capacity preceding myocardial infarction or sudden cardiac death [2,3,4]. Elevated VE is associated with a 1.5- to 2-fold increased risk of future cardiac events [3,4]. In addition to its prognostic role, VE is highly prevalent among patients with established CVD, with reported rates ranging from 35% to 75% [5,6,7,8]. Elevated levels of VE have been documented following myocardial infarction and coronary artery bypass graft surgery, underscoring its relevance across a broad spectrum of CVD [6,8].

Given its high prevalence and prognostic significance in these populations, VE has been identified as an independent risk factor for disease progression [3,4]. More specifically, it is an independent predictor of morbidity and mortality in CVD and is associated with a two- to three-fold higher risk of subsequent cardiac events (e.g., myocardial infarction, repeat revascularization, recurrent angina, re-hospitalization) [5]. Importantly, the consistent association between VE and coronary heart disease has prompted suggestions for its inclusion in European Society of Cardiology (ESC) guidelines [4].

However, the etiology of VE remains incompletely understood and is thought to reflect impaired adaptation to chronic stress, a central mechanism in CVD. Lifestyle factors (e.g., physical inactivity, disordered eating, and alcohol use) and psychosocial stressors (e.g., low social support, occupational strain) may contribute to its development [2,3,4]. At a biological level, VE appears to involve dysregulation of key systems, including the hypothalamic–pituitary–adrenal (HPA) axis, the autonomic nervous system, and inflammatory pathways. Altered cortisol secretion patterns indicate impaired stress adaptation, while reduced heart rate variability reflects autonomic imbalance. In parallel, elevated inflammatory markers such as C-reactive protein, together with indices of fibrinolytic and myocardial stress (e.g., troponin and N-terminal pro–B-type natriuretic peptide), suggest ongoing systemic and cardiovascular strain [4,8,9]. The above mechanisms provide a biologically plausible framework linking VE with anxiety and insomnia. These constructs may mutually reinforce each other, highlighting the clinical relevance of their interrelationship. This interaction may be driven by shared stress-related neurobiological mechanisms, including autonomic dysregulation and impaired stress recovery, and may further aggravate stress responses, fatigue, impaired recovery, and adverse cardiovascular outcomes [10]. Consistent with this framework, the evidence suggests a bidirectional relationship between anxiety and insomnia, whereby each condition may contribute to the onset and persistence of the other [10].

Importantly, anxiety and sleep disturbances are frequent among cardiac populations. More specifically, insomnia has been reported in approximately 44% of patients with CVD [11]. Anxiety symptoms affect more than one in four inpatients, while anxiety disorders are present in nearly one in ten individuals [12]. In patients with CVD, anxiety ranges from 70% to 80% following acute myocardial infarction and persists long-term in approximately 20–25% of cases [13]. Both anxiety and insomnia have important clinical consequences in patients with CVD. Elevated anxiety levels are associated with prolonged hospitalization, increased healthcare costs, and a higher risk of readmission [14], while insomnia is linked to an increased risk of all-cause mortality over long-term follow-up [11].

From a clinical perspective, the coexistence of VE, anxiety, and insomnia may represent a significant burden in hospitalized patients with CVD, adversely affecting patient outcomes, recovery, and prognosis. From a research perspective, the integration of these interrelated factors remains underexplored, as they have largely been investigated separately, and without sufficient consideration of their combined effects. Improved understanding of these relationships may facilitate the early identification of vulnerable patients and support targeted interventions, ultimately enhancing patient management and reducing hospital length of stay and healthcare costs. These findings have implications at both the patient level and the healthcare system level. At the patient level, they may support risk stratification and individualized care strategies. At the healthcare system level, they may inform the development of hospital-wide strategies and policy frameworks aimed at improving the quality and efficiency of care delivery.

To the best of our knowledge, this is one of the first studies to simultaneously examine VE, anxiety, and insomnia in hospitalized patients with CVD and to explore the associations of these factors with demographic and clinical characteristics. This integrated approach extends previous research by addressing these interrelated conditions within a single clinical population rather than in isolation, providing a more comprehensive understanding of their coexistence. By highlighting the clinical relevance of VE, anxiety, and insomnia, the findings of this study may contribute to improved awareness and recognition of VE in clinical practice.

Accordingly, the present study aimed to explore the association of VE with anxiety and insomnia in hospitalized patients with CVD, as well as the demographic and clinical characteristics associated with VE.

## 2. Materials and Methods

### 2.1. Design, Setting, and Period of the Study

This single-center, cross-sectional study was conducted at a public hospital in Athens, Greece, between December 2024 and December 2025. A total of 200 hospitalized patients with CVD were included. Participants were recruited using a convenience sampling approach, and the study aimed to explore associations between the investigated variables at a single time-point.

The final sample size was determined based on all eligible and consenting patients available during the study period; therefore, a formal a priori power calculation was not performed. During the study period, 250 hospitalized patients with CVD were assessed for eligibility. Of these, 240 met the inclusion criteria and were invited to participate. Forty patients either declined participation, did not provide informed consent, or returned incomplete questionnaires, resulting in a final analytical sample of 200 participants.

### 2.2. Inclusion and Exclusion Criteria of the Sample

The inclusion criteria were as follows: (i) age ≥ 18 years; (ii) ability to read, write, and understand the Greek language; and (iii) a confirmed diagnosis of CVD. The exclusion criteria included: (i) hospitalization primarily for conditions other than CVD and (ii) cognitive impairment or significant visual or hearing deficits, documented in the medical records, that could impair participation in the study. The diagnosis of CVD was confirmed through review of physician-documented diagnoses in the hospital electronic health system.

### 2.3. Data Collection and Procedure

Recruitment was conducted by the primary researcher using a convenience sampling approach during the study period. Eligible patients were identified through daily review of hospital admission lists and medical records. Patients who met the inclusion criteria were approached during their hospital stay, informed about the purpose of the study, and invited to participate. Written informed consent was obtained prior to enrollment. Data were collected through structured face-to-face interviews conducted by the researcher, using the study’s instruments. Data collection was scheduled during evening shifts, when patients were not undergoing medical procedures, diagnostic tests, or clinical interventions, in order to ensure a stable and standardized environment for interview completion. All participants were assessed under the same time conditions, which ensured consistency in data collection across the sample and reduced variability related to different assessment times. Each interview lasted approximately 30 min per participant. Given the interview-based data collection approach, any incomplete responses were handled using listwise deletion and excluded from the final analysis.

### 2.4. Research Instruments

Data were collected by the completion of a research instrument which included participants’ characteristics and the following scales: (a) the Maastricht Vital Exhaustion Questionnaire (MEQ), (b) The Zung Self-Rating Anxiety Scale (SAS) and (c) the Athens Insomnia Scale (AIS). The following demographic characteristics were recorded: gender, age, marital status, educational level, and occupation. Clinical characteristics included type of disease, years since disease onset, and the presence of comorbidities. In addition, patients’ perceptions regarding whether they felt well-informed about their health and whether they considered written information to be important were assessed.

#### 2.4.1. Measurement of VE

The MEQ, originally developed by Appels and Mulder, is a psychometric instrument designed to assess VE [1]. The scale consists of 20 items designed to measure individuals’ VE. Each item is rated on a three-point response scale (Yes, No, and I don’t know). All items load on a single factor after reverse scoring of items 9 and 14. The total score ranges from 0 to 40, with higher scores indicating higher levels of VE [1,15]. The MEQ has demonstrated high reliability and validity in the Greek population [15].

#### 2.4.2. Measurement of Anxiety

Anxiety was evaluated using the SAS, a widely used and validated instrument designed to assess both psychological and somatic symptoms of anxiety. The scale comprises 20 items reflecting the respondent’s experiences over the past week, with responses rated on a four-point Likert scale (1–4) according to symptom frequency. Selected items are reverse-scored to enhance the accuracy of the total score. The total anxiety score is calculated by summing individual item responses, with higher scores indicating greater symptom severity [16]. For the Greek-language version, the SAS scale has demonstrated satisfactory reliability and validity [17].

#### 2.4.3. Measurement of Insomnia

The AIS was used to assess insomnia. The scale consists of eight items evaluating nighttime sleep and daytime mood. Each item is rated on a 0–3 scale, with the total score obtained by summing all items, yielding a range from 0 to 24. Higher scores indicate greater severity of insomnia. The scale has been translated into the Greek language, and shows high reliability and validity [18].

### 2.5. Ethical Considerations

The present study was approved by the Research Committee of the relevant public hospital (Decision of the eighth meeting of the Council, 27 November 2024). The study was conducted in accordance with the ethical standards of the Declaration of Helsinki (World Medical Association, 1989). Patients who met the inclusion criteria were informed by the researcher about the aims and procedures of the study. Written informed consent was obtained from all participants prior to enrolment. Participation was voluntary, and participants were informed of their right to refuse or withdraw from the study at any time without any consequences. Data collection ensured the anonymity and confidentiality of all participants.

### 2.6. Statistical Analysis

Categorical variables are presented as absolute and relative frequencies (%), while continuous variables are presented as mean and ± standard deviation, or median and interquartile range (IQR). The normality of continuous variables was assessed using the Kolmogorov–Smirnov test, in addition to visual depictions using histograms and Q–Q plots. Associations between scale scores and patient characteristics were examined using the Kruskal–Wallis test, the Mann–Whitney U test, and Spearman’s rho correlation coefficient, as appropriate. Subsequently, multiple linear regression analysis was performed to estimate the effects of patient characteristics (independent variables) on VE scores (dependent variable). Results are presented as β coefficients with 95% confidence intervals. Statistical significance was set at *p* < 0.05. All statistical analyses were performed with SPSS version 28 (SPSS Inc., Chicago, IL, USA).

## 3. Results

### 3.1. Sample Description

Table 1 presents the description of the CVD sample.

The majority were men (60.7%), 71–80 years old (40%), of high school education (58.6%), and retired (68.6%), who had angina (30.7%), and were at the time of CVD diagnosis (35%). A majority of patients had a comorbidity (91.4%), declared themselves to be well-informed about heart problems (49.3%) and considered the receiving of written information to be important (65.7%).

### 3.2. Measurement of VE and Anxiety and Insomnia

Table 2 shows that the mean VE score was 21.4 ± 10.1. Based on the possible score range (0–40), these values indicate moderate levels of VE. Regarding anxiety, the mean anxiety score was 49.9 ± 10.4. Considering the possible score range (20–80), these values reflect moderate levels of anxiety. In terms of insomnia, the mean score was 8.2 ± 5.7. Based on the possible score range (0–24), these findings indicate moderate severity.

The Cronbach’s α coefficients indicated high internal consistency for VE (0.885), SAS (0.827), and AIS (0.927).

### 3.3. Factors Associated with VE

Table 3 presents the associations of patients’ characteristics with VE. Patients’ VE was found to be statistically significantly associated with gender (*p* = 0.002), age (*p* = 0.006), occupation (*p* = 0.006), disease type (*p* < 0.001), whether they had comorbidity (*p* = 0.001) and how important they considered receiving written information (*p* = 0.015).

More specifically, female patients exhibited higher VE scores (median: 18) compared with male patients (median: 10). Likewise, patients aged > 80 years showed higher VE scores than those aged < 60 years, 61–70 years, and 71–80 years (medians: 9, 14, and 12, respectively). Similarly, unemployed patients presented higher VE scores (median: 24) compared with employed (median: 9) and retired patients (median: 14). Accordingly, patients with heart failure demonstrated higher VE scores (median: 20) than those with angina (median: 10), myocardial infarction (median: 12), or atrial fibrillation (median: 13). In addition, patients with comorbid conditions exhibited higher VE scores (median: 14) compared with those without comorbidities (median: 5). Finally, patients who did not consider receiving written information important at all reported lower VE scores (median: 8) compared with those who considered it very important (median: 14).

### 3.4. Correlation of VE with Anxiety and Insomnia Scores

Table 4 presents the correlations between patients’ VE scores and anxiety, as well as for insomnia. All correlations were statistically significant (*p* < 0.05). The correlations were positive (rho > 0.2), indicating that an increase in VE score implied increases in the insomnia and anxiety scores.

### 3.5. Multiple Linear Regression Analysis of VE

Multiple linear regression was performed to assess the effects of characteristics that were found to be statistically significantly associated with the patients’ VE. The results are presented in Table 5 (Model 1).

It was found that female patients had 2.5 points-higher VE score compared to male patients (β = 2.5 95%CI: [0.2–4.7], *p* = 0.032). All patients in the age groups over 60 years had greater VE, compared to patients under 60 years (age group 61–70: β = 6.1 95%CI: [2.5–9.8], *p* = 0.001; age group 71–80: β = 3.6 95%CI: [0.5–6.7], *p* = 0.023; and age group > 80: β = 4.4 95%CI: [1.0–7.9], *p* = 0.013). Patients with atrial fibrillation or angina had 5.4 and 3.6 units less VE than patients with heart failure (β = −5.4 95%CI: [−8.3–−2.6], *p* = 0.001 and β = −3.6 95%CI: [−6.4–−0.7], *p* = 0.014, respectively). Patients who considered it of little or no importance to receive written information had 3.8 and 3.1 units less VE than patients who considered it very important (β = −3.8 95%CI: [−7.3–−0.3], *p* = 0.035 and β = −3.1 95%CI: [−5.6–−0.6], *p* = 0.016, respectively). Furthermore, an increase in the scores of trait anxiety or insomnia by one unit implies an increase in the score of VE by 0.4 and 0.6 units, respectively (β = 0.4 95%CI: [0.3–0.5], *p* = 0.001 and β = 0.6 95%CI: [0.4–0.8], *p* = 0.001, respectively).

A second model was performed (Table 5–Model 2), removing the insomnia and anxiety scores, to check if patient characteristics remained significant. We observed that, after this removal, age and importance of written information were no longer significantly associated with VE.

## 4. Discussion

According to the results of the present study, associations were shown between VE and insomnia, anxiety and clinical and demographic characteristics in hospitalized patients with CVD; the study’s objectives were thereby addressed. Given the cross-sectional design of the study, it cannot be determined whether VE levels pre-existed hospitalization, potentially contributing to hospital admission, or developed during the hospital stay.

The following sections discuss these findings in the context of the existing literature and explore potential mechanisms that may underlie the observed associations.

The higher VE observed in women is consistent with previous studies. Bechsgaard et al. [19] reported higher VE levels in women with chest pain and no obstructive coronary artery disease, compared with asymptomatic women, suggesting that symptom burden may contribute to elevated VE. A study conducted in Italy [20] similarly found a high prevalence of VE among women enrolled in cardiac rehabilitation, where it was associated with psychological distress, insomnia, and adverse lifestyle and psychosocial factors. In patients with acute myocardial infarction prior to cardiac rehabilitation, women have also been shown to experience higher levels of anxiety, VE, and sleep disturbances [8]. These sex-related differences are clinically relevant, as women are less likely to participate in cardiac rehabilitation programs [8], potentially limiting access to secondary prevention strategies. These findings may be understood in the context of sex-specific differences in stress reactivity and psychosocial burden, including greater exposure to caregiving responsibilities and socioeconomic stressors among women. Such factors may contribute to sustained activation of neurobiological stress systems, including the hypothalamic–pituitary–adrenal axis and inflammatory pathways, thereby increasing vulnerability to both psychological distress and cardiovascular dysregulation. Τhese data suggest a consistent pattern of greater psychological and symptom-based burden among women with CVD, likely driven by the interaction between biological stress mechanisms and psychosocial factors [21].

Furthermore, the present findings indicated higher VE among unemployed participants, suggesting a potential link between VE and socioeconomic disadvantage. Socioeconomic status is a multidimensional construct that differentiates segments of society based on access to resources (financial, educational, and material) as well as patterns of living conditions, including occupational class, housing quality, and residential environment [22]. Within this context, employment status represents a key indicator of individuals’ access to economic and social resources and is therefore closely linked to adverse health outcomes. In line with the present findings, a prior study among 362 patients referred for coronary angiography reported that lower income and educational level were associated with higher levels of VE, independent of disease severity and functional status [23].

Accordingly, the results indicated higher VE among patients with heart failure compared with those with angina, myocardial infarction, and atrial fibrillation. This observation may be explained by the high prevalence of fatigue in heart failure, a core component of VE, which has been reported in 70–80% of patients and substantially impairs daily functioning, including self-care activities. The underlying mechanisms are likely multifactorial and include reduced cardiac output, impaired oxygen delivery to peripheral tissues, decreased skeletal muscle strength, and dysregulation of the autonomic nervous system. In addition, patients with heart failure are often older, present with multiple comorbidities, and have reduced social support networks, all of which may contribute to increased vulnerability to VE [24,25]. Supporting this evidence, the ARIC study, which included 14,348 participants followed for a median of 16.9 years, demonstrated that greater social isolation was associated with an increased risk of incident heart failure hospitalization or mortality, independent of demographic factors, lifestyle behaviors, and comorbidities [26].

The higher VE observed among older participants and those with comorbidities may reflect the cumulative impact of aging and multimorbidity on physical functioning and psychological well-being in patients with cardiovascular disease. These conditions are associated with impaired functional status, reduced energy levels, poorer quality of life, and increased mortality [27], factors that may increase vulnerability to the development and persistence of VE.

An encouraging finding of the present study is that patients with VE considered written health information to be important, suggesting receptiveness to educational interventions. One possible explanation is that the fatigue and emotional distress characterizing VE may reduce patients’ ability to retain verbal information, increasing the value of written educational materials. Such resources may reinforce understanding, support self-management, and facilitate adherence to clinical recommendations. Furthermore, well-informed patients are more likely to communicate effectively with healthcare professionals and participate in shared decision-making [28].

Higher VE scores were associated with increased anxiety and insomnia. In patients with acute myocardial infarction, elevated VE has been linked to higher levels of anxiety and depression, associations that persist after adjustment for demographic and clinical characteristics [6,7]. Anxiety is often accompanied by sleep disturbances, while insomnia may further exacerbate anxiety through reduced restorative sleep. This reciprocal relationship may contribute to reduced energy levels and the development or maintenance of VE. Given that daytime fatigue is a core feature of VE, persistent sleep disturbances may increase vulnerability to exhaustion, reduce psychological resilience, and impair stress coping capacity [29,30]. Overall, these mechanisms may help explain the observed interrelationship between VE, anxiety, and insomnia.

The interrelationship between VE, anxiety, and insomnia in hospitalized patients may be interpreted in terms of environmental, psychological, and pharmacological factors. Apart from the primary disease, inpatients are faced with multiple psychosocial stressors, including limited family contact, unfamiliar hospital environments, exposure of intimate aspects of care to unfamiliar individuals or medical equipment, financial concerns, uncertainty regarding disease progression or treatment, and challenges in interactions with healthcare staff [31,32]. Furthermore, environmental factors such as noise, artificial lighting, and frequent staff interventions may predispose patients to sleep disturbances. In particular, hospitalization is associated with disruption of normal circadian rhythms, leading to fragmented sleep architecture, with approximately half of total sleep occurring during daytime hours [33].

Several potential confounding factors should be considered in the interpretation of the present findings. VE substantially overlaps with depressive symptoms, which may complicate the differentiation between these constructs; however, previous evidence suggests that VE remains an independent predictor of recurrent vascular events after adjustment for relevant confounders, whereas depressive symptoms, anxiety, and hostility do not, supporting its distinct clinical role [34]. In addition, VE shares conceptual similarities with burnout, particularly in relation to chronic stress exposure [35]. Lifestyle-related factors, including smoking, physical inactivity, and alcohol consumption, may also influence VE levels and should be considered as potential confounders [36]. Chronic pain has been associated with increased psychological stress, and a higher risk of sleep disturbances [37]. Data collection was conducted only during evening hours, which may represent a potential confounding factor, as fatigue levels and psychological symptoms may vary throughout the day, potentially influencing participants’ responses. Furthermore, the high prevalence of comorbidities (91.4%) in the present sample reflects the clinical complexity of hospitalized patients and may have influenced the observed associations. The predominantly male composition of the sample (60.7%), together with evidence of higher VE levels in women, suggests that sex may also represent an important factor influencing the interpretation of the findings. Finally, in the sensitivity analysis (Model 2), after excluding insomnia and anxiety scores, age and the importance of written information were no longer significantly associated with VE. This finding suggests that the observed associations in the primary model may be influenced by psychological symptom burden, acting either as a potential confounder or as part of an underlying pathway linking patient characteristics with VE. However, given the cross-sectional design, no causal inferences can be made.

### 4.1. Clinical Implications

In clinical practice, the present findings suggest that VE is associated with sex, age, socioeconomic status, comorbidities, disease type, perceived importance of written health information, and psychological factors, including anxiety and insomnia, in patients with CVD. These subgroups may benefit from closer clinical monitoring and more individualized assessment strategies. Furthermore, the observed associations highlight the potential value of educational interventions tailored to enhance patient understanding and engagement in care. Overall, these findings underscore the clinical complexity of VE in cardiovascular populations and support the need for comprehensive assessment of both psychological and somatic factors in routine practice.

### 4.2. Limitations of the Study

Interpretation of these results should be made with caution due to certain study limitations. The present study employed a cross-sectional design; therefore, no causal relationships between the variables under investigation can be inferred. It would be of interest to explore whether VE pre-exists CVD or develops as a consequence of it. Furthermore, a second assessment at hospital discharge or at home might reveal potential changes in the studied variables. The use of convenience sampling in a single hospital in Attica limits the generalizability of the findings to the broader population of hospitalized patients with CVD in Greece, while a potential center effect may also have influenced the observed results. Several potential confounding factors were not assessed, factors which may have influenced levels of VE and related outcomes. Moreover, the interview-based data collection approach does not exclude the possibility of interviewer-related bias, which may arise from question wording, non-verbal cues, or researcher expectations. In addition, as self-report instruments were used, recall bias and subjective interpretation should be considered as potential sources of measurement bias. Finally, the relatively small sample size should be acknowledged, although statistically significant associations were observed.

## 5. Conclusions

In conclusion, VE is a clinically relevant condition in hospitalized patients with CVD, showing associations with key demographic and clinical risk profiles as well as with anxiety and insomnia. These findings highlight the importance of recognizing VE as part of a broader psychological and somatic vulnerability pattern in cardiovascular care.

Future research should prioritize large, randomly selected samples and methodological approaches that account for the multifactorial etiology of VE, with particular attention to confounding and mediating factors. Given the observed associations between VE, anxiety, and insomnia, further studies are needed to clarify their combined impact on clinical outcomes. In addition, clinical trials including patients with VE are warranted that evaluate targeted interventions and improve treatment strategies in cardiovascular populations.

## Figures and Tables

**Table 1:** Distribution of the sample according to their characteristics (*n* = 200).

	*n* (%)
Gender	
Male	121 (60.7%)
Female	79 (39.3%)
Age (years)	
≤60	34 (17.1%)
61–70	33 (16.4%)
71–80	80 (40.0%)
>80	53 (26.4%)
Marital status	
Married	116 (57.9%)
Single	20 (10.0%)
Divorced	11 (5.7%)
Widow	53 (26.4%)
Educational level	
Elementary	51 (25.7%)
High school	117 (58.6%)
University	32 (15.7%)
Occupation	
Unemployed	20 (10.0%)
Employed	43 (21.4%)
Retired	137 (68.6%)
Disease	
Heart failure	54 (27.1%)
Myocardial infarction	36 (17.9%)
Atrial fibrillation	49 (24.3%)
Angina	61 (30.7%)
Onset of CVD	
Time of diagnosis	70 (35.0%)
≤1 year	30 (15.0%)
2–5 years	41 (20.7%)
6–10 years	17 (8.6%)
10–15 years	9 (4.3%)
>15 years	33 (16.4%)
Comorbidity	
Yes	183 (91.4%)
No	17 (8.6%)
Informed about the health problem	
Very	99 (49.3%)
Enough	43 (21.4%)
A little	49 (24.3%)
Not at all	9 (5.0%)
Is it important to receive written information	
Very	131 (65.7%)
Enough	6 (2.9%)
A little	17 (8.6%)
Not at all	46 (22.9%)

**Table 2:** Description of questionnaire scores (*n* = 200).

Scores	Score Range	Mean (SD)
Vital Exhaustion (VE)	0–40	21.4 (10.1)
Anxiety (SAS)	20–80	49.9 (10.4)
Insomnia (AIS)	0–24	8.2 (5.7)

SD: Standard deviation.

**Table 3:** Factors associated with VE in CVD patients.

	VE Score		
	Mean (SD)	Median (IQR)	*p*-Value
Gender			0.002
Male	13.4 (9.7)	10 (6–18)	
Female	18.4 (10.1)	18 (12–24)	
Age (years)			0.006
≤60	11.8 (11.7)	9 (4–14)	
61–70	17.7 (9.6)	14 (8–26)	
71–80	14.5 (10.6)	12 (6–22)	
>80	17.5 (7.8)	18 (12–22)	
Marital status			0.071
Married	13.9 (8.8)	12 (6–20)	
Single/Divorced	15.3 (12.6)	10 (6–24)	
Widow	18.8 (10.7)	14 (12–24)	
Educational level			0.728
Primary	14.3 (9.6)	14 (6–23)	
High school	15.4 (10.2)	14 (8–22)	
University	17.0 (11.0)	14 (10–18)	
Occupation			0.006
Unemployed	21.7 (11.3)	24 (16–28)	
Employed	12.4 (11.3)	9 (4–14)	
Retired	15.4 (9.2)	14 (8–20)	
Disease			<0.001
Heart failure	22.6 (10.6)	20 (14–32)	
Myocardial infarction	14.7 (9.1)	12 (8–18)	
Atrial fibrillation	12.3 (8.0)	13 (4–18)	
Angina	11.8 (8.6)	10 (6–14)	
Years since onset of health problem			0.076
Diagnosis	12.5 (9.0)	12 (6–18)	
≤1 year	16.0 (13.7)	10 (6–22)	
2–5 years	18.6 (11.2)	20 (8–26)	
6–15 years	16.9 (8.8)	15 (12–22)	
>15 years	15.6 (6.7)	16 (10–22)	
Comorbidity			0.001
Yes	16.2 (10.1)	14 (8–23)	
No	6.2 (4.2)	5 (2–9)	
Informed about the health problem			0.417
Very	14.7 (10.7)	12 (6–22)	
Enough	16.3 (9.8)	18 (8–24)	
A little/Not at all	15.8 (9.5)	14 (10–20)	
Is important to receive written information?			0.015
Very/Enough	16.8 (10.0)	14 (10–24)	
A little	14.0 (10.0)	14 (5–19)	
Not at all	11.8 (9.8)	8 (6–14)	

SD: Standard deviation; IQR: Interquartile range.

**Table 4:** Correlation between questionnaire scores.

	VE Score	
	Spearman’s Rho	*p*-Value
AIS Score	0.687	<0.001
SAS Score	0.429	<0.001

**Table 5:** Assessment of the impact of factors on patients’ VE.

	VE Score			
	Model 1		Model 2	
	β Coef (95% CI)	*p*-Value	β Coef (95% CI)	*p*-Value
Gender (Females vs. Males)	2.5 (0.2–4.7)	0.032	4.1 (0.6–7.4)	0.020
Age (years)				
≤60	Ref.Cat.		Ref.Cat.	
61–70	6.1 (2.5–9.8)	0.001	2.3 (−3.1–7.7)	0.392
71–80	3.6 (0.5–6.7)	0.023	−0.6 (−5.1–3.9)	0.801
>80	4.4 (1.0–7.9)	0.013	0.9 (−4.1–5.9)	0.718
Disease				
Heart failure	Ref.Cat.		Ref.Cat.	
Heart attack	−1.8 (−5.0–1.3)	0.252	−6.2 (−10.8–−1.6)	0.009
Atrial fibrillation	−5.4 (−8.3–−2.6)	0.001	−10.3 (−14.4–−6.1)	0.001
Angina	−3.6 (−6.4–−0.7)	0.014	−9.1 (−13.1–−5.1)	0.001
Comorbidity (No vs. Yes)	−1.9 (−5.8–1.9)	0.313	−6.3 (−11.9–−0.7)	0.028
Is important to receive written information?				
Very/Enough	Ref. Cat.		Ref. Cat.	
A little	−3.8 (−7.3–−0.3)	0.035	−4.6 (−9.9–0.8)	0.091
Not at all	−3.1 (−5.6–−0.6)	0.016	−2.9 (−6.5–0.7)	0.114
Insomnia score (AIS)	0.6 (0.4–0.8)	0.001	-	
Anxiety score (SAS)	0.4 (0.3–0.5)	0.001	-	

β coeff: β regression coefficients, CI: confidence interval, Ref. Cat.: Reference category. Model includes factors that were not causing multicollinearity effects.

## Data Availability

The datasets generated during the current study are available from the corresponding author on reasonable request.
